# Plagiarism in scientific writing: words or ideas?

**DOI:** 10.3325/cmj.2011.52.576

**Published:** 2011-08

**Authors:** Farrokh Habibzadeh, Karen Shashok

Plagiarism refers to the act of “appropriation of another person’s ideas, processes, results, or words without giving appropriate credit” (1). Most academic researchers reach a consensus that plagiarism is a serious breach of publication ethics (2). Plagiarism has different forms but can be categorized into two general distinct categories – plagiarism of ideas and plagiarism of text (verbatim). No doubt, plagiarism of ideas is a blatant act of misconduct. Plagiarism of text and recycling of words are also a serious fault in humanities and literature where the essence of work and novelty are wordings and eloquence of the text (1). But, what about science where the essence of the work is the originality of the scientific content no matter how eloquent it is presented (3).

The aims of scientific journals are somewhat different from those of non-scientific journals. For example, medical journals are published to promote medicine and betterment of the public health through dissemination of results of scientific research. In many fields such as literature and humanities, different authors may have dissimilar views and attitudes toward a unique situation. Using their talents, through appropriately selected words, the authors try to reflect what they feel – their understanding and the affect they perceive. Therefore, each single word and its collocation would be very important in transferring the feeling to readers and how one would construe from the text. In scientific writing, on the other hand, the researcher whose audience are scholars looking for pure evidence-based facts, should mainly act as no more than a good observer and reporter. Unlike an author in the field of literature, the author of a scientific paper should follow certain well-established scientific methodology and always be careful not to be affected by his or her intuition or different sorts of biases that might jeopardize the judgment of a researcher. In this way, as long as the author is a fair observer and relies on the solid evidence, facts, and well-established scientific methods, no matter how eloquent he or she is, the scientific findings can be reported and published provided that he or she uses a universally well-accepted scientific methodology for conducting and reporting science. As a matter of fact, while in many fields like literature, the author and hence the wordings are the most important part of the article, in scientific writing the scientific content is more important than the author and wordings as long as the text is comprehensible, no matter if it is written by a layperson or a well-educated first-class eloquent author (3). Here, the originality is not in wordings; it is in the scientific content. In fact in many scientific writing courses we advise authors to convey the message in its simplest form – which is usually not its most eloquent form, since science itself, is complex enough and there is no need for sure to make it more complex using sophisticated writing (4). Especially since many of the audience of scientific articles are not native English speakers.

Duplicate publication and redundant publication are misconduct and waste of resources (5). “Readers deserve original content, and merely recycling parts of previously published work constitutes, at best, academic laziness” (2). Though it is completely true for many fields like literature, we are not pretty sure if it is also applicable to science. “Readers” of scientific papers are just looking for science presented in an appropriate format (wordings, graphs, tables, layout, etc). We are not sure if they even care how well the words are used as long as they can understand what the author meant to convey. With enough scrutiny, you can find many typographical and grammatical errors in articles published in even prestigious mainstream journals; in most instances, most of the text can be written in more eloquent forms.

If the originality of a scientific article is not in its wordings but is in its content, why should not one insert a piece of well-written phrase or even sentences (not ideas) from a previously published paper in his or her manuscript to better express him/herself because he or she is disinclined to sacrifice quality and accuracy of the statements either for want of linguistic expertise or “academic laziness” (2). Obviously, it is a must that the author who does so should understand and interpret the original text correctly. As recently suggested, the damage to the integrity of the literature and seriousness of the misconduct associated with text plagiarism are less obvious compared to the consequences of plagiarism of idea (6).

Although many initiatives, say *AuthorAID*, would help non-English speaking authors express themselves acceptably (7), the future would be completely different. Soon, we will have machine translation with enough quality to be used in online versions of scientific journals. The translation machinery is certainly very premature yet to translate efficiently the text in the field of literature – novels, dramas, poems, etc – but it is proficient enough in most cases to preserve the scientific content of scientific articles. Some of the algorithms used by these machines are so that they would result in text similarities in the translated texts. On the other hand, in how many ways can you describe how you take a blood sample or analyze it? If we still insist on preventing text similarities (even our own previous texts – self-plagiarism) in scientific writing, we have to think about inventing new words so we would fool the software programs used for checking plagiarism!

## References

[1] Vessal K, Habibzadeh F. (2007). Rules of the game of scientific writing: fair play and plagiarism. Lancet.

[2] Kleinert S (2011). Checking for plagiarism, duplicate publication, and text recycling.. Lancet.

[3] Habibzadeh F (2010). Judge the article, not the author.. Croat Med J.

[4] Lang TA (2011). The illusion of certainty and the certainty of illusion: a caution when reading scientific articles. Int J Occup Environ Med.

[5] Habibzadeh F, Winker MA (2009). Duplicate publication and plagiarism: causes and cures.. Notfall und Rettungsmedizin..

[6] Steen RG (2011). Retractions in the scientific literature: do authors deliberately commit research fraud?. J Med Ethics.

[7] Shashok K (2009). Editing around the world: AuthorAID in the Eastern Mediterranean: A communication bridge between mainstream and emerging research communities.. European Science Editing.

